# The Relationship between Moral Intelligence and Value Conflict Resolution Ability in University Students

**Published:** 2020-02

**Authors:** Maryam AKBARILAKEH, Lili MOHANDES MOJARRAD, Shahram YAZDANI, Leila AFSHAR, Alireza MORAVEJI

**Affiliations:** 1. Department of Medical Education, School of Management and Medical Education Sciences, Shahid Beheshti University of Medical Sciences, Tehran, Iran; 2. Department of Medical Ethics, School of Traditional Medicine, Shahid Beheshti University of Medical Sciences, Tehran, Iran; 3. Department of Community Medicine, School of Medicine, Social Determinants of Health Research Center, Kashan University of Medical Sciences, Kashan, Iran

## Dear Editor-in-Chief

Conflict is an integral part of human life and it's quite natural. Throughout history, it always accompanied by human life (1). If these disagreements do not manage correctly and scientifically, they can cause problems, issues and even violence and oppression (2).

Values are created as a value system inside the person and the basis for judging and deciding on different affairs. Values by creating attitudes cause behavioral orientation (3).

Resolving and management ability value conflicts require skills. These skills have basic and emotional core and have decisive role in student’s educational outcome. Moral intelligence as a kind of directional finder is acting right and has ability to apply universal ethics interacting with others. It looks like; enhancing moral intelligence can strengthen the ability of the management to value the conflict.

Based on the theory of formation and moral development have an impact on the nature of perfection, potential good clean ability from bad, human motivations and emotions, growth of thought and cognitive judgment, the objective and behavioral experiences of the individual and the social environment, his cultural and educational influence on moral development. In this regard, the factor of the educational environment has a mutual relationship and the ability to influence other factors (4).

The first principle is that we judge matters with values and decide on the same basis. The values give us an attitude that is the basis of our movement and our behavior. The second principle is that values are not a separate concept. They are put in the form of a value system. Therefore, we judge a set of values about things and act on the same basis (5). The value source in some societies is wisdom and personal inference. In other words, values are created by their minds; it means, what they find themselves have value (6). This study was performed with the goal of identifying the relationship between moral intelligence and ability to resolve (manage) value conflicts in Kashan University of Medical Science students, Kashan, central Iran in 2017. It was correlated with other studies (7–9) conflict resolution questionnaire, as research tool.

On research tools, factor analysis tests were conducted and the average Cronbach's alpha coefficient 0.711∼0.791 and composite reliability coefficient 0.822∼0.825 were computed respectively for moral intelligence questionnaire and conflict resolution capability questionnaire. Their reliability has been confirmed. Due to considering the population of the surveyed population as well as the contribution of the educational grades, the sample size was selected by using Cochran formula and included 245 people.

This study was approved at Shahid Beheshti University of Medical Sciences with the code of ethics IR.SBMU.RETECH.REC.1396.954.

Mean earned moral intelligence rating by studied medical students was equal to 66.29 which was in the poor level in terms of moral intelligence category. They Embracing Responsibility for Serving Others index was the least (6.47) and Admitting Mistakes and Failures index (6.75) earned the most average. Average earned score of conflict resolution capability by studied medical students was 112.27 which placed in the medium level in terms of the classification of conflict resolution ability score. Participating medical students in the study view conflict as natural and positive index was 12.13. The highest rate, as well as the Develop “DOABLES,” Stepping-stones to Action index were (11.25) and got the lowest average score.

The results of this study showed the relationship between the amounts of moral intelligence with the ability to manage conflict resolution value in medical students studying in the 2016–2017 academic year in Kashan University of Medical Sciences. They have positive and significant relationship (0.867) with an error level 0.01.

The coefficient of determination for simple regression was calculated 0.721 based on the results of simple regression analysis. In other words, Moral Intelligence explained 72% of variance in Conflict Resolution Ability (R^2^= 72).

Moreover, no significant difference between the amount of moral intelligence and the ability rate to manage value conflicts in medical students with demographic characteristics (age, gender, number of children in the family, history of nerve disease, marital status and educational level) was observed.

Findings of this research need to review the educational curriculum as well as the attention of teachers to rapid change and evolution training demands raising the level of learners’ moral intelligence. Therefore, a valued training course to raise the level of moral intelligence and naturally, the ability to resolve the conflict of value of medical students must be considered.
